# Management of Patients with Atrial Fibrillation: Specific Considerations for the Old Age

**DOI:** 10.4061/2011/854205

**Published:** 2011-08-11

**Authors:** Laurent M. Haegeli, Firat Duru

**Affiliations:** Clinic of Cardiology, Cardiovascular Center, University Hospital of Zurich, Rämistrasse 100, 8091 Zurich, Switzerland

## Abstract

Atrial fibrillation (AF) is the commonest of all sustained arrhythmias, and most of the patients seeking medical therapy are in the elderly age group. The management of these patients is particularly difficult due to associated comorbidities. Hypertension, congestive heart failure, left ventricular hypertrophy, and coronary artery disease are often present in the elderly patient population, and therefore, antiarrhythmic drugs often fail due to side effects, proarrhythmia, or poor rhythm control. Recently, radiofrequency catheter ablation has been widely performed as an efficient therapy for recurrent, drug-refractory AF. Nevertheless, patients at old age were underrepresented in prior AF ablation trials, and the current guidelines for catheter ablation of AF recommend a noninvasive approach in the elderly patient group due to the lack of clinical data supporting ablation therapy. However, study results of our group and others are suggesting that catheter ablation is a safe and effective treatment for patients over the age of 65 years with symptomatic, drug-refractory AF, and therefore, patients should not be precluded from catheter ablation only on the basis of age. This paper discusses the pharmacological (rhythm control, rate control, and anticoagulation) and catheter management of AF in the elderly population.

## 1. Introduction

**Figure 1:**
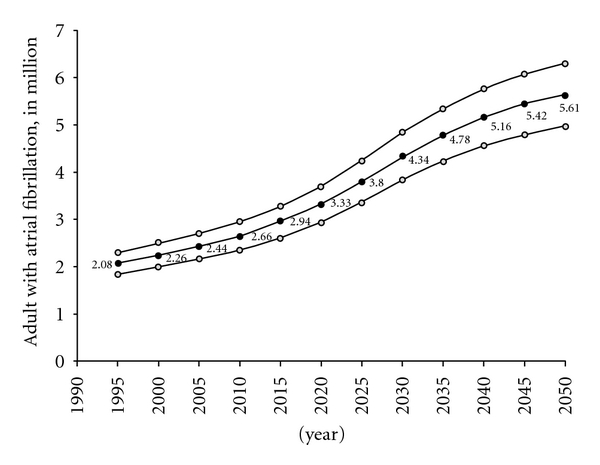
Projected number of adults with AF in the United States between 1995 and 2050 from the ATRIA study (the AnTicoagulation and Risk Factors in Atrial Fibrillation Study) [5].

Atrial fibrillation (AF) is the commonest of all sustained arrhythmias, and its prevalence has been increasing. AF confers an important mortality and morbidity outcome from thromboembolism, stroke, heart failure, and significant impairment of quality of life [1, 2]. The prevalence of AF is more prominent with advanced age. About 8 to 10% of people aged over 80 years are suffering from AF [3]. The median age of an AF patient is 75 years, and 70% of the AF patients are 65 to 85 years of age [4, 5]. Within the next twenty to thirty years, the number of patients suffering from AF is expected to double or triple due to an increased AF incidence and aging of the populations in developed western countries (Figure 1) [5, 6].

In former days, the management of AF focused on preventing thromboembolism and controlling heart rate or rhythm. The risk of stroke is increased 5-fold by AF. AF is responsible for around 10–20% of all strokes. In patients age groups 80 to 89 years, this proportion is even more accentuated and around 25% [7]. Strokes related to AF result often in higher mortality and morbidity rates. The use of oral anticoagulation therapy is an important intervention in preventing AF-related ischemic events. But older people have both higher risk for stroke if not taking oral anticoagulants and higher risk for bleeding with the use of oral anticoagulants [8]. Therefore, the recommendation for anticoagulation is a challenging task for the clinician treating patients with AF in the old age. Disease management is also particularly challenged by comorbidities including hypertension, congestive heart failure, left ventricular hypertrophy, coronary artery disease, and diabetes mellitus which are frequently present in this patient age group. These comorbidities also confer an increased risk for thromboembolic complications or drug-related side effects [9]. Moreover, other endpoints such as left ventricular and atrial function, quality of life, social functioning, silent cerebral embolism and dementia are novel targets of comprehensive AF disease management [10].

Ablation therapy has emerged as an efficient intervention for recurrent, drug-refractory AF [11–14]. Current ablation techniques have improved, and the complication rates have decreased resulting in increasing number of referrals of patients of old age for catheter ablation of AF [15–18]. Nevertheless, a minority of elderly patients were included in prior AF ablation trials. Friable cardiac structures, which may be at risk for catheter perforation, long procedure times, and the associated comorbidities, are frequently considered to confer an increase of overall peri- and postprocedural risk. For that reason and in the absence of clinical data, the recommendation in the guidelines for catheter ablation of AF advices a conservative approach in patient populations of old age [19]. Nevertheless, with advanced life expectancy, the elderly population group is a rapidly expanding portion of our community making AF an even more important public health concern. Catheter ablation could become a pivotal treatment strategy in the elderly patient population after failure of antiarrhythmic drugs.

## 2. Stroke Prevention in the Elderly

Oral vitamin K antagonists efficiently reduce the risk of cerebrovascular embolism in elderly AF patients as clearly shown in several randomized trials [20, 21]. Patients with AF aged over 75 years have a thromboembolic complication risk of over 4% per year, mandating therapy with oral vitamin K antagonists unless there is a significant risk for major bleeding present. Among each components of the widespread CHADS_2_ (cardiac failure, hypertension, age, diabetes, and stroke (doubled)) risk score, age ≥ 75 years confers an impaired prognosis for stroke and mortality over hypertension, heart failure, or diabetes [22]. Therefore, the CHADS_2_ score was extended recently to the CHA_2_DS_2_-VASc score by considering additional risk factors such as vascular disease (i.e., prior myocardial infarction), age between 65 and 74 years, and female sex (Table 1) [23]. The risk for stroke can be reduced by oral vitamin K antagonists by about 70% and consecutively the mortality by 33% [24]. But, these agents have a small therapeutic window with an associated hemorrhagic risk complicating anticoagulation management. In general, the anticoagulation intensity should be optimized by keeping the international normalized ratio (INR) between 2.0 and 3.0 [25]. Several studies have shown that low fixed-dose use of an oral vitamin K antagonist or targeting lower INRs (<2.0) in older patient groups increase the risk for stroke without protecting against intracerebral bleeding [25–27]. In cases where oral vitamin K antagonists are contraindicated, antiplatelet therapy with aspirin provides some prevention from cerebrovascular embolism, but much less efficiently than oral vitamin K antagonists [28]. Aspirin reduces the risk for stroke by about 20%. Interestingly, the beneficial effect of antiplatelet therapy on ischemic stroke appears to diminish with increasing age and is no longer present after the age of 77 years [29, 30]. Warfarin was found to be superior to combined therapy with clopidogrel plus aspirin with similar rates of bleeding complications in the Atrial fibrillation Clopidogrel Trial with Irbesartan for prevention of Vascular Events (ACTIVE-W) study [31]. A novel generation of oral anticoagulants is emerging and being approved for AF such as dabigatran, an oral direct thrombin inhibitor. In a large randomized trial (Randomized Evaluation of Long-Term Anticoagulation Therapy; RE-LY), dabigatran has shown to be superior to warfarin in terms of similar reduction of stroke rates, but lower rates for major bleeding [32]. Apixaban, a novel factor Xa inhibitor, was superior to aspirin for reduction in stroke without increase of major bleeding in 5599 patients (mean age of 70 years), who are unsuitable for vitamin K antagonist therapy, as reported in the AVERROES trial [33]. Elderly patients are less likely than younger patients to receive appropriate anticoagulation and are more likely to have subtherapeutic INR levels. In general practice, fewer than half of eligible patients take warfarin [34, 35]. High fall risk, history of bleeding, nonadherence, and dementia are the major factors preventing physicians to prescribe oral anticoagulants [36–38]. Therefore, in elderly patients ineligible for vitamin K antagonist therapy, oral direct thrombin or factor Xa inhibitor, dabigatran, or apixaban, respectively, should be considered as an effective and safe option.

## 3. Pharmacological Management in the Elderly

Several randomized trials comparing rhythm control versus rate control in AF patients showed no evidence that the clinical outcome of hospitalization, stroke, and mortality is improved by restoration and maintenance of sinus rhythm despite the clear relationship between AF and cardiovascular events [39–44]. Moreover, rhythm control by pharmacological interventions has been associated with higher mortality in the elderly [40]. However, subgroup analyses and the recent published outcome data of the ATHENA trial signalize that safely maintained sinus rhythm by novel antiarrhythmic drugs may prevent AF-related complications [45, 46]. This placebo-controlled, double-blinded conducted study assessed the efficacy of dronedarone for the prevention of cardiovascular hospitalisation or death from any cause in patients with AF and atrial flutter. Nevertheless, apart from the effect of dronedarone on the composite endpoint driven by cardiovascular hospitalizations in the ATHENA trial, there are no controlled data available that show a benefit of rhythm control therapy beyond improved quality of life. The major studies on rhythm versus rate control were the rate control versus electrical cardioversion (RACE) trial [39], the atrial fibrillation follow-up investigation of rhythm management (AFFIRM) trial [40], and the atrial fibrillation congestive heart failure (AF-CHF) trial [43]. There was also a series of smaller studies performed, including the pharmacological intervention in atrial fibrillation (PIAF) [44], strategies of treatment of atrial fibrillation (STAF) [41], and how to treat chronic atrial fibrillation (HOT CAFÉ) [42]. These studies have shown that primary rate control is not inferior to rhythm control. Therefore, first-line therapy in the elderly patient population with symptomatic AF is usually a primary rate control approach. Betablockers, nondihydropyridine calcium channel blockers, and digoxin are widely used to control the ventricular rate response in AF [35]. Digoxin can be added if impaired left ventricular systolic function is present, but caution should be raised because of potential drug toxicity, especially in elderly patients with frequent impaired renal function and polypharmacy. Previous guidelines recommended targeting a resting heart rate of less than 80 beats per minute. But a recent randomized trial showed no clinical benefit of a strict rate control versus a lenient rate control targeting resting heart rates of about 115 beats per minute in terms of clinical cardiovascular events [47]. Antiarrhythmic drugs with the aim to maintain sinus rhythm may be considered, if patients remain symptomatic despite optimal rate control, but the increased risk for proarrhythmia, drug interactions, and age-related comorbidities in the elderly population should be carefully taken into account. Class Ic antiarrhythmic drugs, flecainide, and propafenone have shown to increase mortality in patients with coronary artery disease [48]. Sotalol and dofetilide should not be used in patients with renal impairment. Amiodarone is the most effective drug and safe in heart failure patients, but regular follow-up of thyroid, hepatic, and pulmonary function is mandatory because of frequent extracardiac drug toxicity. Therefore, amiodarone should be reserved for use if other antiarrhythmic drugs have failed or are contraindicated.

## 4. Catheter Ablation of AF in the Elderly

An effective alternative option for drug-refractory AF with a rapid ventricular rate response is the transvenous catheter ablation of the atrioventricular node and the placement of a permanent pacemaker. The procedure is associated with minimal mortality and morbidity, but this approach does not eliminate AF and the need for anticoagulation [49]. Pathophysiological knowledge that focal sources of ectopic beats arising from the pulmonary veins often initiate AF has lead to the development of catheter ablation for AF in the last decade [11]. The majority of ablation strategies currently used involves circumferential ablation around the ostia of the ipsilateral pulmonary veins with the endpoint of electrical isolation of the pulmonary veins from the left atrium [50–52]. Success rates approach 70% to 90% in experienced centers [53]. However, most of the published data are obtained in younger patients aged below 65 years and without heart disease and comorbidities. Catheter ablation for chronic AF is less successful than for paroxysmal AF and is associated with higher complication rates in older patients having structural heart disease [14, 54, 55]. Procedure-related complication rates were reported in a large worldwide multicenter survey and are listed on Table 2 [17]. In a retrospective analysis of 641 consecutive ablation procedures, the rate for major complications was 5%, and the age greater than 70 years was identified as a significant predictor with an odds ratio of 3.7 [18].

**Figure 2:**
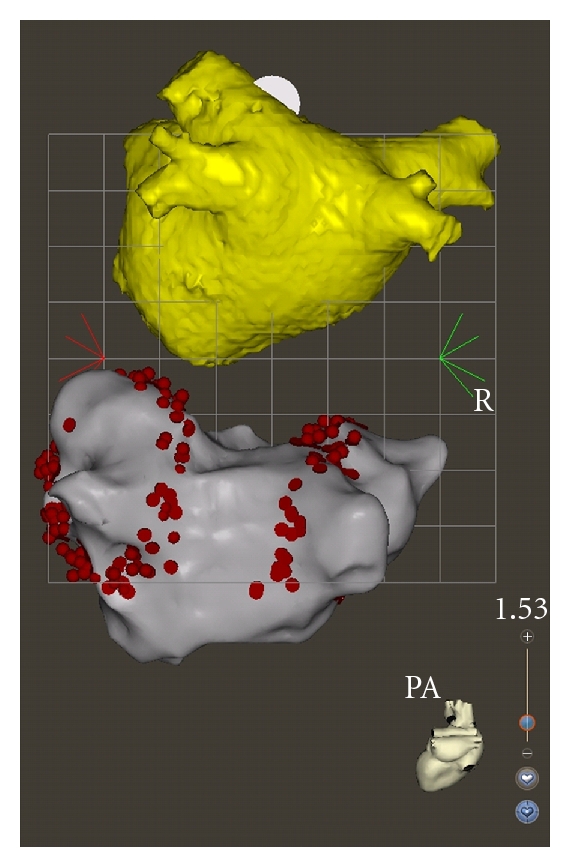
Three-dimensional reconstruction (yellow) of the computed tomography imaging and three-dimensional electro-anatomical map (grey) of the left atrium in posteroanterior projection with circumferential ablation (red points) around ipsilateral pulmonary veins using CARTO system (Biosense Webster Inc., Diamond Bar, Calif, USA).

**Figure 3:**
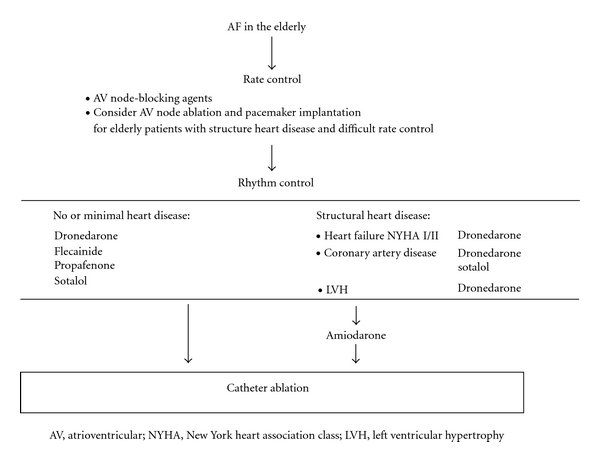
Decision tree for the therapy of AF in the elderly integrating pharmacological treatment and catheter ablation, modified from the guidelines for the management of AF proposed by the European Society of Cardiology [63].

In a recently published study, we reported the clinical outcome of 45 consecutive patients over the age of 65 years who underwent a percutaneous catheter ablation procedure for symptomatic paroxysmal and persistent AF [57]. Among them, none had a significant structural heart disease. All patients underwent wide-area circumferential pulmonary vein isolation for paroxysmal AF with additional linear lesions for persistent AF. The ablation was performed point by point by radiofrequency energy and guided by a three-dimensional electroanatomical mapping system (Figure 2) [61]. The endpoint of the procedure in both paroxysmal and persistent AF patients was electrical isolation of all pulmonary veins, which was assessed using a circular spiral catheter. Our results suggested that catheter ablation of AF in elderly patients can be performed with success rates comparable to those in younger patients without an increase in complication rate. Successful maintenance of a stable sinus rhythm could be achieved in nearly 80% of this patient cohort with a mean age of 69 years (Table 3). Zado et al. found similar success and complication rates in patients over 65 years of age [60]. Patients over the age of 80 years in the paper of Tan et al. were less likely to undergo a repeat procedure than younger patients. However, the success and complication rates were not significantly different in the age group over 80 years than in those 60–69 years (70% versus 74% for success rate) [59]. Similarly, the study reported by Bunch et al. found no increased risk of periprocedural complications in patients aged 80 years and older [58]. Available published outcome data for catheter ablation in the elderly population were derived from observational cohort analysis with a follow-up period of up to two years with procedural success defined as freedom from symptomatic AF. A long-term follow-up study reported that the success rate in 100 patients was 63% at 5 years after a median of two procedures per patient [62]. Prospective, randomized trials comparing an invasive versus a conservative pharmacological approach are required to address the remaining questions on best management of AF in the elderly population. A decision tree integrating different choices of rate and rhythm control and pharmacological therapy versus catheter ablation of AF in this selected elderly patient population is proposed in Figure 3 based on the current guidelines of the European Society of Cardiology [63].

## 5. Conclusion

Elderly patients differ considerably from patients in the younger age group as they have a higher incidence of AF associated with a higher thromboembolic risk due to advanced age and frequent multiple comorbidities. In addition, the adverse side effects of antiarrhythmic drugs, such as proarrhythmia, are more commonly observed in the elderly patient population. Nonrandomized studies in patients aged 65 years and more with symptomatic drug-refractory AF have shown that catheter ablation can be performed with comparable safety and efficiency as with younger patients. Therefore, ablation therapy may be considered as an appropriate therapeutic option also for the older group of patients if antiarrhythmic drug treatment fails. Patients should not be precluded from undergoing AF catheter ablation exclusively on the basis of age.

## Figures and Tables

**Table 1:** Stroke risk assessment in nonvalvular AF: CHA_2_DS_2_-VASc [23]. For a CHA_2_DS_2_-VASc score > 1, such patients are high risk, and oral anticoagulation is recommended; for a CHA_2_DS_2_-VASc = 1, either oral anticoagulation or apirin 75 to 325 mg daily is recommended, but oral anticoagulation is preferred rather than aspirin; for a CHA_2_DS_2_-VASc = 0, either aspirin 75 to 325 mg daily or no antithrombotic treatment can be used, but no antithrombotic therapy is preferred.

Risk factors	Score
Congestive heart failure/LV dysfunction	1
Hypertension	1
Age ≥ 75 years	2
Diabetes mellitus	1
Stroke/TIA/TE	2
Vascular disease (prior MI, PAD, or aortic plaque)	1
Age 65–74 years	1
Sex category (i.e., female sex)	1

Maximum score	9

TIA: transient ischemic attack; TE: thromboembolic event.

**Table 2:** Major complication rates in a worldwide survey of catheter ablation for AF in 16,309 patients from Cappato et al. [56].

Type of complication	Rate, %
Death	0.15
Tamponade	1.31
Pneumothorax	0.09
Hemothorax	0.02
Sepsis	0.01
Phrenic nerve palsy	0.17
Femoral pseudoaneurysm	0.93
Arteriovenous fistulae	0.545
Valve damage/requiring surgery	0.07
Atrio-esophageal fistulae	0.04
Stroke	0.23
Transient ischemic attack	0.71
PV stenoses requiring intervention	0.29

Total	**4.54**

PV: pulmonary vein.

**Table 3:** Catheter ablation of AF in the elderly.

	Haegeli et al. [57]	Bunch et al. [58]	Tan et al. [59]	Zado et al. [60]
Inclusion age (years)	≥65	≥80	≥80	65–74
			70–79	≥75
			60–69	
Mean age (years)	69 ± 3.5	82 ± 2	84 ± 5	68 ± 3
			75 ± 4	77 ± 2
			66 ± 4	
Number of patients	45	35	49	185
			151	32
			177	
Number of procedures	53	35	53	228
			174	34
			209	
Paroxysmal AF (%)	87	46	55	62
			53	53
			51	
Ablation strategy	PVI ± linear	PVI ± linear	PVI	PVI
	lesions	Lesions		
Mean F/U (months)	6	12	18	27
Periprocedural complication rate (%)				
(i) Pericardial tamponade	1.9	2.8	0.2	0.4
(ii) Deep venous thrombosis	0	2.8	0.9	0
(iii) CVA/TIA	0	0	0.7	0.8
(iv) Retroperitoneal bleeding	0	0	0.7	0.4
(v) Pseudoaneurysm/AV fistula	030	0	0.5	2.7
Freedom of AF	74%	78%	70%	84%
			72%	86%
			74%	

PVI: pulmonary vein isolation; CVA: cerebral vascular accident; TIA: transient ischemic attack.
